# A comparison of the five-minute cognitive test with the mini-mental state examination in the elderly for cognitive impairment screening

**DOI:** 10.3389/fnins.2023.1146552

**Published:** 2023-06-09

**Authors:** Xiaoli Pan, Xiaoqin Cheng, Jie Zhang, Yingfeng Xia, Chunjiu Zhong, Guoqiang Fei

**Affiliations:** ^1^Department of Neurology, Zhongshan Hospital (Xiamen Branch), Fudan University, Xiamen, Fujian, China; ^2^Department of Neurology, Zhongshan Hospital, Fudan University, Shanghai, China; ^3^State Key Laboratory of Medical Neurobiology, Institutes of Brain Science and Collaborative Innovation Center for Brain Science, Fudan University, Shanghai, China

**Keywords:** mild cognitive impairment, Alzheimer’s disease, reliable and valid cognitive screening test, MMSE, magnetic resonance imaging

## Abstract

The five-minute cognitive test (FCT) is a novel cognitive screening method with the quick and reliable merit for detecting cognitive impairment at an early stage. The diagnostic power of FCT in differentiating subjects with cognitive impairment from people with cognition in a normal range was demonstrated effective as that of the Mini-Mental Status Evaluation (MMSE) in a previous cohort study. Here, we analyzed the effect of sociodemographic and health-related factors on FCT performance and further investigated the consistency of FCT. Then, we compared the correlation of subitem scores of FCT or MMSE with a comprehensive battery of neuropsychological tests that focus on specific domains of cognition. Finally, the association of the total FCT scores with the volumes of brain subregions was investigated. There were 360 subjects aged 60 years or above enrolled in this study, including 226 adults with cognitive abilities in normal range, 107 subjects with mild cognitive impairment (MCI) and 27 mild Alzheimer’s disease (AD). The results showed that the total FCT scores was negatively associated with increasing age (*β* = −0.146, *p* < 0.001), and positively associated with education attainment (*β* = 0.318, *p* < 0.001), dwelling condition with family (*β* = 0.153, *p* < 0.001) and the Body Mass Index (*β* = 1.519, *p* < 0.01). The internal consistency of the FCT (Cronbach’s *α*) was 0.644. The sub-scores of FCT showed a significant correlation with other specific neuropsychological tests. Impressively, the total FCT scores showed a significantly positive association with the volumes of hippocampus related subregions (*r* = 0.523, *p* < 0.001) and amygdala (*r* = 0.479, *p* < 0.001), but not with cerebellum (*r* = 0.158, *p* > 0.05) or subcortical subregions (*r* = 0.070, *p* > 0.05). Combining with previous data, FCT is a reliable and valid cognitive screening test for detecting cognitive impairment in a community setting.

## Introduction

Dementia is a major cause of disability in older adults which affects memory and other cognitive abilities such as attention, executive function and changes in mood and behaviors. According to the report from World Health Organization, over 55 million people live with dementia worldwide and the number was predicted to rise to 78 million by 2030 (World Health Organization, 2021). In 2019, dementia was the seventh leading cause of death worldwide, and the associated cost was estimated to be more than $1.3 trillion which causes a huge economic and mental burden for individual family and the society (2022 Alzheimer's Disease Facts and Figures, 2022).

Unfortunately, over 60% patients live in low- and middle-income countries and lack of adequate medical service, leading to the dilemma that most patients cannot be diagnosed at the early stage of dementia (World Health Organization, 2021). Since more than one third of global dementia cases may be preventable by changing or modifying lifestyle factors to reduce the risk (Livingston et al., 2017), it is urgent to detect the cases with cognitive impairment at the preclinical or pre-dementia stage. A reliable cognitive screening test with the quick and convenient merits will ensure early recognition of cognitive deficit and timely preventive intervention especially in a large-scale epidemiological study (Lonie et al., 2009; Tsoi et al., 2015).

The five-minute cognitive test (FCT) designed by Zhang J was utilized to capture deficits in a multi-domain of cognitive function, including episodic memory, language fluency, time orientation, visuospatial, and executive function (Zhang et al., 2019). The FCT scores range from 0 to 20 and lower scores reflect worse cognitive performance. The average completion time of FCT was 339.9 ± 67.7 s (5–6 min) in old adults. The impaired episodic memory has been reported as the early symptom and found to predict progression to AD among subjects with mild cognitive impairment (MCI) (Eckerstrom et al., 2013; Sala et al., 2017). Therefore, FCT was designed with greater emphasis on assessing episodic memory (8 points). The diagnostic power of FCT in differentiating patients with cognitive impairment from people with cognitive function in a normal range was reported effective as that of the Mini-Mental Status Evaluation (MMSE; Folstein et al., 1975) in the previous cohort study (Zhang et al., 2019).

Here, we analyzed the sociodemographic and health-related factors on FCT performance in a larger sample sized population and further investigated the consistency of FCT. Then, the correlation of subitem scores of FCT or MMSE with a comprehensive battery of neuropsychological tests representing specific cognitive domains was studied to compare the capacity of FCT and MMSE for assessing cognitive impairment. Finally, the association of total FCT scores with the volume of different brain subregions were analyzed.

## Methods

The study design and procedures were approved by the Committee on Medical Ethics of Zhongshan Hospital, Fudan University.

### Study population

Total 360 subjects were enrolled from the unban community of Xuhui District, Shanghai, China, from March, 2016 to March, 2018. There were 226 individuals with cognitive abilities in normal range, 107 patients with MCI and 27 patients with mild AD. All subjects were over 60 years old and have an educational background over 6 years. Written informed consents were obtained from all participants or the authorized caregivers. The information of epidemiological data, history of disease, current medications and lifestyle were collected by questionnaire.

The exclusion criteria were: (1) severely visual and hearing impairments and unable to complete neuropsychological tests and scale assessments; (2) suffered from severe central nervous system diseases or mental illness; (3) Alcohol or drug abusers; (4) Other physical disease may impair cognitive function; (5) Participated in other clinical trials within 6 months.

Subjects with MMSE scores over 24 points and 0 score of Clinical Dementia Rating (CDR; Morris, 1993) were recognized in normal group. The definition of MCI was obtaining a score of 24 or higher on the MMSE, a score of 0 or 0.5 on CDR, a subjective cognitive complaint, objective cognitive impairment as examined by a comprehensive battery of neuropsychological assessments, essentially preserved activities of daily living, and were not demented (Petersen et al., 1999; Petersen, 2004). The mild AD met the National Institute of Neurological and Communicative Disorders and Stroke and AD and Related Disorders Association criteria for probable AD (McKhann et al., 1984) and had a score of 0.5 or 1 on the CDR.

### Neuropsychological and clinical assessments

All the participants received FCT, MMSE and CDR assessment to determine the general cognitive function and daily life ability was assessed by the Activities of Daily Living scale (ADL). The subitem scores of different domains of FCT and MMSE were listed. The subjects in normal control and MCI group further underwent a comprehensive battery of neuropsychological tests for assessing specific cognitive domains, such as memory, language fluency, visuospatial ability, attention and executive function. The scales include the Auditory Verbal Learning Test (AVLT; Guo and Hong, 2001) for memory test, Animal Fluency Test (AFT; Zhao et al., 2007) and Boston Naming Test (BNT-30; Rabin et al., 2005) for language ability, Trail Making Test A and B (TMT-A, TMT-B; Lu et al., 2006) and Symbol Digit Modalities Test (SDMT; Smith, 1982) for attention and executive function, and the Rey-Osterrieth Complex Figure Test (CFT; Guo and Hong, 2000) for visuospatial ability.

### MRI data acquisition and analysis

Participants were scanned by a 3.0 Tesla Siemens Verio MRI scanner in Zhongshan Hospital, Fudan University. One hundred and 76 contiguous sagittal T1 weighted high-resolution structural images were obtained by using 3D spoiled perturbed gradient echo sequence and the parameters were as follows: TR/TE: 1,900/2.5 ms, flip angle: 9, slice thickness 1 mm, field of view 256 mm, voxel resolution 1 × 1 × 1 mm, 8-channels head receiver coil. Each sequence scan lasted for 4 min and 18 s. CAT12 was utilized to segment T1 data and to estimate registration parameters to convert the probability maps into MNI152NLin2009cAsym space. Then the volume of hippocampus related fields, amygdala, subcortical structures and the cerebellum was separately extracted from each subject according to The Cobra atlas, which was built from four atlases provided by the Computational Brain Anatomy Laboratory at the Douglas Institute (CoBra Lab; Entis et al., 2012; Zhang et al., 2019).

### Statistical analysis

Statistical analyses were performed using SPSS software (version 21.0; IBM SPSS). All *p* values were two-sided with statistical significance level set at <0.05. The results were expressed as mean ± SD for demographic data. Kruskal–Wallis test or Pearson chi-square test was used to compare demographic data among groups of control subjects, MCI and mild AD. The cognitive assay data were compared between control subjects and MCI was using Student’s t-test or nonparametric Mann–Whitney *U* test for continuous variables. The internal consistency of the five subdomains of FCT was analyzed using Cronbach’s α test. Multiple linear regression model was used to analyze the effect of sociodemographic and health-related factors on FCT and MMSE total scores as well as the sub-scores. Linear regression was used to evaluate the relationship between subitem scores of FCT or MMSE and cognitive performance assayed by AVLT, ANF, Boston-30, TMT-A/B, SDMT and CFT, respectively. Spearman correlation analysis was utilized between the total FCT scores and the volume of different brain subregions.

## Results

### Demographic data

The detailed demographic data were shown in Table 1. We recruited 226 subjects with normal cognition (NC), 107 subjects with MCI and 27 patients with mild AD in this study. The mild AD group was the oldest and has the lowest education attainment as compared with those in NC and MCI groups (age: 72.00 ± 7.90 in AD, 69.67 ± 5.97 in MCI, 68.09 ± 5.43 in NC, *p* < 0.01; education: 9.89 ± 4.14 in AD, 11.36 ± 2.90 in MCI, 12.30 ± 2.91 in NC, *p* < 0.0001). The Body Mass Index (BMI) in NC group was larger than that in mild AD group (23.91 ± 3.59 vs. 21.77 ± 3.31, *p* < 0.05). No significant difference was found in gender, dwelling condition (live with family or not), prevalence of hypertension, diabetes mellitus or hyperlipidemia among the three groups (Table 1).

**Table 1:** Demographic characteristics of participants (mean ± SD).

	Control	MCI	Mild AD	*p* value
Characteristics	*n* = 226	*n* = 107	*n* = 27	
Age (year)	68.09 ± 5.43	69.67 ± 5.97	72.00 ± 7.90	**<0.01**
Gender n (female %)	142 (62.8)	68 (63.6)	17 (63.0)	0.992
Education (year)	12.30 ± 2.91	11.36 ± 2.90	9.89 ± 4.14	**<0.0001**
BMI (Kg/m2)	23.91 ± 3.59	23.39 ± 4.60	21.77 ± 3.31	**<0.05**
Hypertension *n* (%)	123 (54.4)	53 (49.5)	12 (44.4)	0.496
Diabetes mellitus *n* (%)	42 (18.6)	28 (26.2)	4 (14.8)	0.207
Hyperlipidemia *n* (%)	99 (43.8)	42 (40.2)	11 (40.7)	0.808
Dwelling with family *n* (%)	207 (91.6)	95 (88.8)	21 (77.8)	0.077
FCT total	17.89 ± 1.14	14.91 ± 2.78	7.98 ± 3.26	**<0.0001**
Delayed recall	6.72 ± 0.77	5.66 ± 1.77	2.09 ± 2.12	**<0.0001**
Language fluency	1.94 ± 0.26	1.65 ± 0.60	1.00 ± 0.74	**<0.0001**
Orientation	2.90 ± 0.30	2.68 ± 0.58	1.96 ± 0.98	**<0.0001**
Drawing	3.46 ± 0.65	2.83 ± 0.92	1.93 ± 1.00	**<0.0001**
Attention and executive function	2.85 ± 0.54	2.08 ± 1.16	1.00 ± 1.18	**<0.0001**
MMSE total	27.89 ± 1.38	26.25 ± 1.87	20.11 ± 2.90	**<0.0001**
Delayed recall	1.96 ± 0.97	1.49 ± 1.06	0.67 ± 0.73	**<0.0001**
Language ability	7.49 ± 0.67	7.22 ± 1.00	5.89 ± 1.01	**<0.0001**
Orientation	9.90 ± 0.32	9.55 ± 0.78	6.89 ± 2.08	**<0.0001**
Drawing	0.99 ± 0.10	0.96 ± 0.13	0.71 ± 0.47	**<0.0001**
Attention and calculation	4.55 ± 0.65	4.19 ± 1.09	3.26 ± 1.46	**<0.0001**
CDR	0.00 ± 0.00	0.36 ± 0.22	0.96 ± 0.13	**<0.0001**
ADL	20.13 ± 0.38	20.84 ± 0.81	24.63 ± 2.90	**<0.0001**
AVLT-delayed recall	5.96 ± 1.91	2.91 ± 2.08	/	**<0.0001**
AFT	16.80 ± 4.16	13.93 ± 3.71	/	**<0.0001**
BNT-30	24.64 ± 3.35	21.67 ± 3.76	/	**<0.0001**
TMT-A	54.93 ± 13.28	74.65 ± 37.11	/	**<0.0001**
TMT-B	146.51 ± 42.46	205.49 ± 67.58	/	**<0.0001**
SDMT	39.23 ± 7.48	30.89 ± 9.33	/	**<0.0001**
CFT	30.40 ± 2.91	26.45 ± 4.72	/	**<0.0001**

MCI: mild cognitive impairment; FCT: five-minute cognitive test; MMSE: mini-mental state examination; CDR: clinical dementia rating; ADL: activities of daily living scale; AVLT: auditory verbal learning test; AFT: animal fluency test; BNT-30: Boston Naming Test-30; TMT-A: trail making test A; TMT-B: trail making test B; SDMT: symbol digit modalities test; CFT: Rey–Osterrieth complex figure test.

### Cognitive performance

The global cognitive function was assayed by FCT and MMSE in the whole samples. There was a trend towards a progressive decrease among the three groups. The mild AD group showed the worst cognitive performance (FCT: 7.98 ± 3.26 in AD vs. 14.91 ± 2.78 in MCI vs. 17.89 ± 1.14 in NC; MMSE: 20.11 ± 2.90 in AD vs. 26.25 ± 1.87 in MCI vs. 27.89 ± 1.38 in NC; both *p* < 0.0001). The average scores of CDR and ADL were also significantly different among the three groups (listed in Table 1). As compared with NC group, MCI showed a comprehensive and significant decline in multi-domain cognitive function: AVLT delayed recall (5.96 ± 1.91 vs. 2.91 ± 2.08), ANF (16.80 ± 4.16 vs. 13.93 ± 3.71) and BNT-30 (24.64 ± 3.35 vs. 21.67 ± 3.76), TMT-A (54.93 ± 13.28 vs. 74.65 ± 37.11), TMT-B (146.51 ± 42.46 vs. 205.49 ± 67.58) and SDMT (39.23 ± 7.48 vs. 30.89 ± 9.33), CFT (30.40 ± 2.91 vs. 26.45 ± 4.72; all above *p* < 0.0001; Table 1).

### Effect of sociodemographic and health-related factors on scores of FCT and MMSE

We analyzed the effect of sociodemographic and health-related factors on FCT and MMSE total scores as well as the subitem scores in the whole samples (*n* = 360). Age was negatively correlated with total FCT (*β* = −0.146, *p* < 0.001) score, total MMSE score (*β* = −0.105, *p* < 0.001) and all subitem scores of the two scales except for the attention & calculation subitem score of MMSE (Table 2). Education attainment was positively associated with total FCT (*β* = 0.318, *p* < 0.001) score, total MMSE score (*β* = 0.263, *p* < 0.001) and all subitem scores of the two scales except for the orientation sub-score of FCT and delayed memory sub-score of MMSE (Table 2). The condition of dwelling with family was positively correlated with total score (*β* = 0.153, *p* < 0.001), sub-score of delayed recall of FCT (*β* = 0.796, *p* < 0.01) and total score (*β* = 1.340, *p* < 0.01), language ability (*β* = 0.471, *p* < 0.01) as well as orientation sub-scores (*β* = 0.529, *p* < 0.01) of MMSE. BMI was positively associated with total FCT score (*β* = 1.519, *p* < 0.01), subitems of delayed recall (*β* = 0.089, *p* < 0.001), language ability (*β* = 0.014, *p* < 0.05) and orientation of FCT (*β* = 0.023, *p* < 0.01) as well as subitem of orientation of MMSE (*β* = 0.051, *p* < 0.001). The comorbidity with hypertension, diabetes, and hyperlipidemia and gender did not show an obvious correlation with total FCT or MMSE scores (Table 2).

**Table 2:** The effect of sociodemographic and health-related factors on cognitive assessment by FCT and MMSE (*n* = 360).

	Age	Gender	Education	Dwelling with family	BMI	Hypertension history	Diabetes history	Hyperlipemia history	*R* ^2^
FCT total	*β* = −0.146 **p < 0.001**	*β* = −0.067 *p* = 0.851	*β* = 0.318 **p < 0.001**	*β* = 0.153 **p < 0.001**	*β* = 1.519 **p < 0.01**	*β* = −0.275 *p* = 0.434	*β* = 0.259 *p* = 0.531	*β* = 0.190 *p* = 0.586	0.182
Delayed recall	β = −0.058 **p < 0.001**	β = −0.024 *p* = 0.903	β = 0.086 **p < 0.01**	β = 0.796 **p < 0.01**	β = 0.089 **p < 0.001**	β = −0.079 *p* = 0.686	β = 0.101 *p* = 0.659	β = 0.236 *p* = 0.221	0.115
Language fluency	β = −0.025 **p < 0.001**	β = −0.034 *p* = 0.553	β = 0.035 **p < 0.001**	β = 0.133 *p* = 0.124	β = 0.014 **p < 0.05**	β = 0.050 *p* = 0.373	β = −0.005 *p* = 0.944	β = −0.009 *p* = 0.869	0.119
Orientation	β = −0.008 ***p* = 0.105**	β = 0.001 *p* = 0.986	β = 0.007 *p* = 0.441	β = 0.156 *p* = 0.089	β = 0.023 **p < 0.01**	β = −0.055 *p* = 0.353	β = 0.035 *p* = 0.619	β = 0.032 *p* = 0.592	0.058
Drawing	β = −0.025 **p < 0.01**	β = −0.095 *p* = 0.328	β = 0.102 **p < 0.001**	β = 0.159 *p* = 0.283	β = 0.009 *p* = 0.442	β = −0.135 *p* = 0.159	β = 0.180 *p* = 0.112	β = 0.028 *p* = 0.771	0.147
Executive function	β = −0.031 **p < 0.01**	β = 0.083 *p* = 0.440	β = 0.088 **p < 0.001**	β = 0.267 *p* = 0.107	β = 0.017 *p* = 0.189	β = −0.054 *p* = 0.612	β = −0.052 *p* = 0.682	β = −0.091 *p* = 0.393	0.109
MMSE total	β = −0.105 **p < 0.001**	β = −0.031 *p* = 0.915	β = 0.263 **p < 0.001**	β = 1.340 **p < 0.01**	β = 0.057 *p* = 0.106	β = −0.021 *p* = 0.943	β = 0.020 *p* = 0.952	β = 0.024 *p* = 0.933	0.149
Delayed recall	β = −0.024 **p < 0.05**	β = −0.152 *p* = 0.216	β = 0.015 *p* = 0.425	β = −0.025 *p* = 0.895	β = −0.003 *p* = 0.866	β = −0.037 *p* = 0.763	β = 0.171 *p* = 0.234	β = 0.070 *p* = 0.562	0.055
Language ability	β = −0.018 **p < 0.05**	β = 0.219 **p < 0.05**	β = 0.098 **p < 0.001**	β = 0.471 **p < 0.01**	β = −0.003 *p* = 0.803	β = −0.005 *p* = 0.958	β = −0.162 *p* = 0.171	β = 0.068 *p* = 0.494	0.130
Orientation	β = −0.031 **p < 0.01**	β = 0.061 *p* = 0.603	β = 0.073 **p < 0.001**	β = 0.529 **p < 0.01**	β = 0.051 **p < 0.001**	β = −0.072 *p* = 0.534	β = −0.003 *p* = 0.984	β = 0.149 *p* = 0.198	0.126
Drawing	β = −0.005 **p < 0.01**	β = −0.008 *p* = 0.676	β = 0.015 **p < 0.001**	β = 0.045 *p* = 0.115	β = 0.002 *p* = 0.413	β = 0.020 *p* = 0.292	β = −0.015 *p* = 0.489	β = −0.010 *p* = 0.571	0.101
Attention and calculation	β = −0.015 *p* = 0.095	β = −0.123 *p* = 0.257	β = 0.039 **p < 0.05**	β = 0.290 *p* = 0.081	β = 0.011 *p* = 0.395	β = 0.156 *p* = 0.150	β = 0.009 p = 0.943	β = −0.221 p < 0.05	0.054

### Reliability analysis

The internal consistency of the FCT (Cronbach’s *α*) was 0.644 (Table 3). The subdomain correlation was high for delayed recall and language fluency (*r* = 0.538, *r* = 0.527, respectively).

**Table 3:** Internal consistency of Cronbach’s alpha of FCT.

	Scale mean if item deleted	Scale variance if item deleted	Corrected item-total correlation	Alpha if item deleted
FCT language fluency	14.4686	9.314	0.527	0.592
FCT orientation	13.4853	9.457	0.447	0.607
FCT drawing	13.0908	8.285	0.416	0.586
FCT executive function	13.7686	7.828	0.436	0.573
FCT delayed recall	10.1944	4.190	0.538	0.598

Cronbach *α* = 0.644 of the five subdomains in the five-minute cognitive test (FCT).

### Correlation between the subitem scores of FCT or MMSE and specific neuropsychological assessments

**Figure 1:**
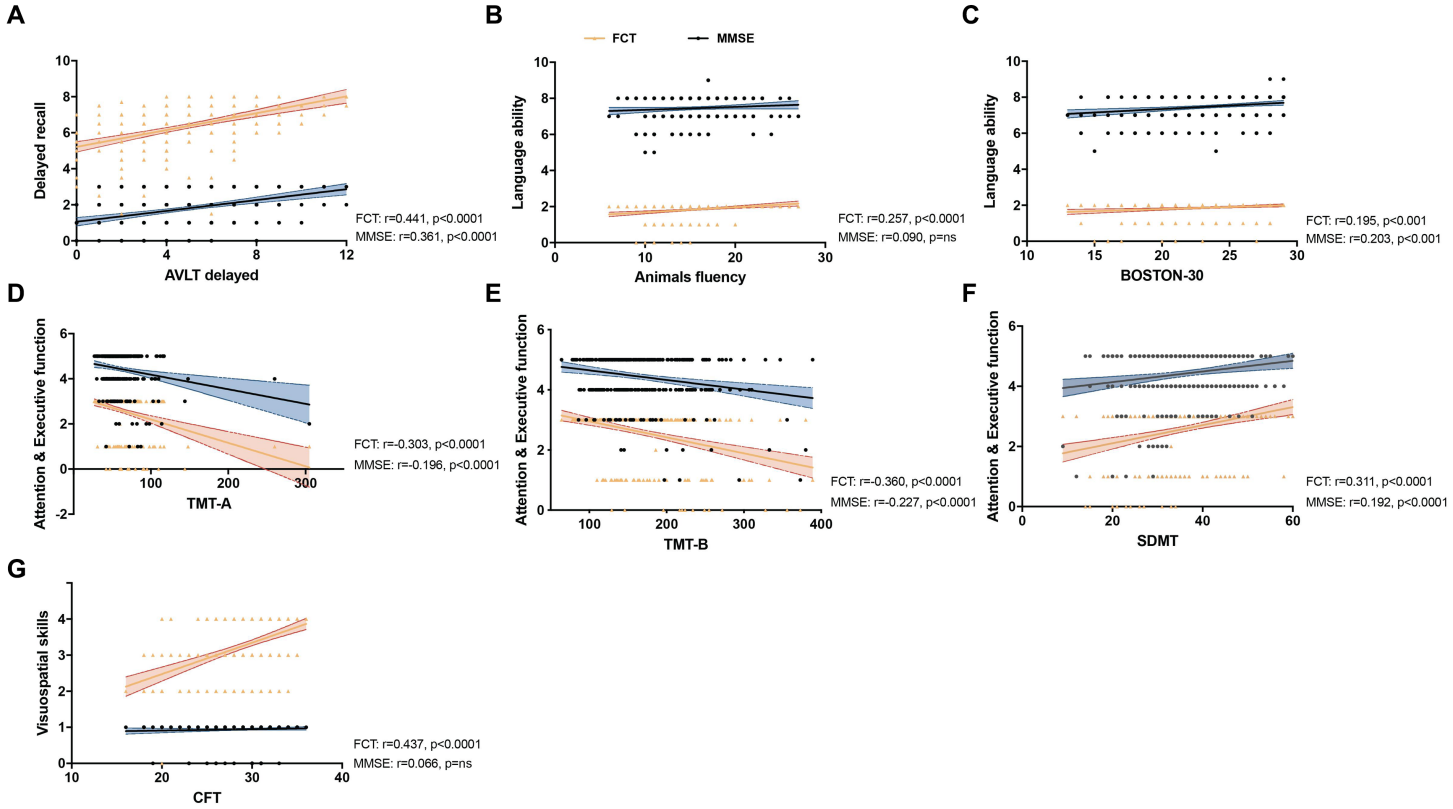
Correlation between the subitem scores of five-minute cognitive test (FCT) or MMSE and specific neuropsychological assessments. Linear regression was used to analysis the correlation of the subitem scores of FCT (Yellow) and MMSE (Blue) with the scores of AVLT delayed **(A)**, Animals fluency (ANF) **(B)**, BOSTON-30 **(C)**, TMT-A **(D)**, TMT-B **(E)**, SDMT **(F)** and CFT **(G)** with different cognitive domain, including delay recall, language ability, attention and executive function and visuospatial skills (*n* = 333). **(A)** The sub-scores of delayed recall of FCT and MMSE both showed a positively correlation with AVLT N5 score (FCT: *r* = 0.441, *p* < 0.0001; MMSE: *r* = 0.361, *p* < 0.0001). **(B)** The ANF score was positively correlated with sub-score of FCT but not MMSE (FCT: *r* = 0.257, *p* < 0.0001; MMSE: *r* = 0.090, *p* = ns). **(C)** BNT was significantly associated with both the sub-scores of FCT and MMSE (FCT: *r* = 0.195, *p* < 0.001; MMSE: *r* = 0.203, *p* < 0.001). **(D–F)**: The sub-scores of FCT and MMSE both demonstrated a significant correlation with TMT-A (FCT: *r* = −0.303, *p* < 0.0001; MMSE: *r* = −0.196, *p* < 0.0001), TMT-B (FCT: *r* = −0.360, *p* < 0.0001; MMSE: *r* = −0.227, *p* < 0.0001) and SDMT (FCT: *r* = 0.311, *p* < 0.0001; MMSE: *r* = 0.192, *p* < 0.0001). **(G)** The sub-score of FCT was positively associated with CFT score but the subitem score of MMSE showed no significant correlation (FCT: *r* = 0.437, *p* < 0.0001; MMSE: *r* = 0.066, *p* = ns).

We further analyzed the correlation of the subitem scores of FCT and MMSE with the scores of specific neuropsychological assessments related to different cognitive domain in NC and MCI groups (*n* = 333). For memory test, the sub-scores of delayed recall of FCT and MMSE both showed a positively correlation with AVLT N5 score (FCT: *r* = 0.441, *p* < 0.0001; MMSE: *r* = 0.361, *p* < 0.0001). For language ability performance, AFT score was positively correlated with the sub-score of FCT but not MMSE (FCT: *r* = 0.257, *p* < 0.0001; MMSE: *r* = 0.090, P = ns). The other indicator for testing language ability, BNT, was significantly associated with both the sub-scores of FCT and MMSE (FCT: *r* = 0.195, *p* < 0.001; MMSE: *r* = 0.203, *p* < 0.001). For attention and executive function, the sub-scores of FCT and MMSE both demonstrated a significant correlation with TMT-A, TMT-B and SDMT (TMT-A: FCT: *r* = −0.303, *p* < 0.0001; MMSE: *r* = −0.196, *p* < 0.0001; TMT-B: FCT: *r* = −0.360, *p* < 0.0001; MMSE: *r* = −0.227, *p* < 0.0001; SDMT: FCT: *r* = 0.311, *p* < 0.0001; MMSE: *r* = 0.192, *p* < 0.0001). For visuospatial assessment, CFT score was positively associated with the sub-score of FCT but not with MMSE sub-scores (FCT: *r* = 0.437, *p* < 0.0001; MMSE: *r* = 0.066, p = ns; Figure 1; Supplementary Table 1).

### Correlation of the total FCT score with the volume of different brain regions

**Figure 2:**
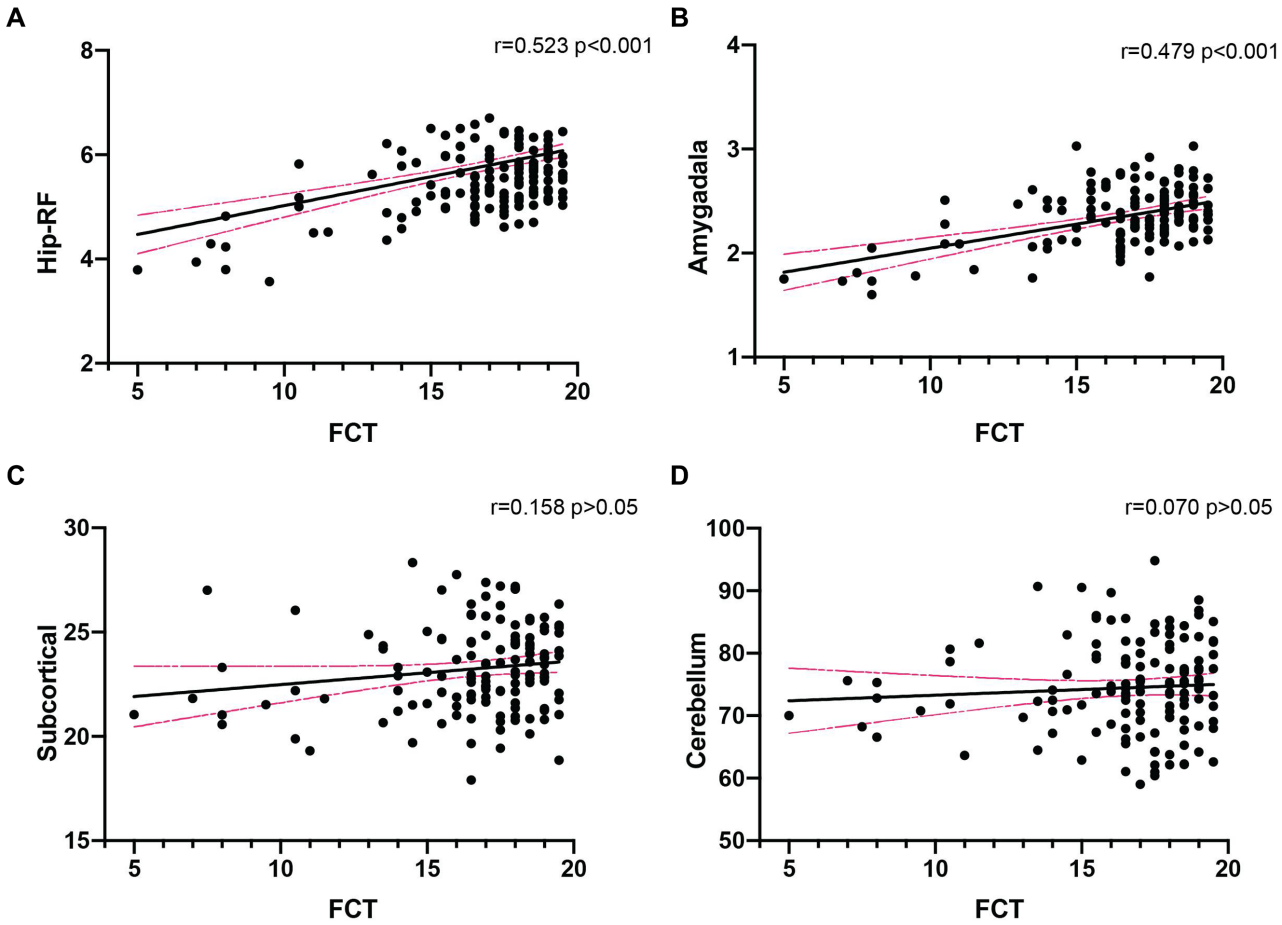
Correlation analysis between FCT scores and the volume of different brain regions in all subjects (*n* = 135). **(A,B)**. FCT scores was significantly associated with the volume of Hip-RF (*r* = 0.523, *p* < 0.001) and amygdala (*r* = 0.479, *p* < 0.001). **(C,D)** There was no significant relationship between FCT scores and the volume of subcortical (*r* = 0.158, *p* > 0.05) or cerebellum (*r* = 0.070, *p* > 0.05). Hip-RF, hippocampus-related regions including hippocampal white matter atlas and subfields. Subcortical, subcortical atlases of the striatum, globus pallidus and thalamus (volume unit: cm3).

**Figure 3:**
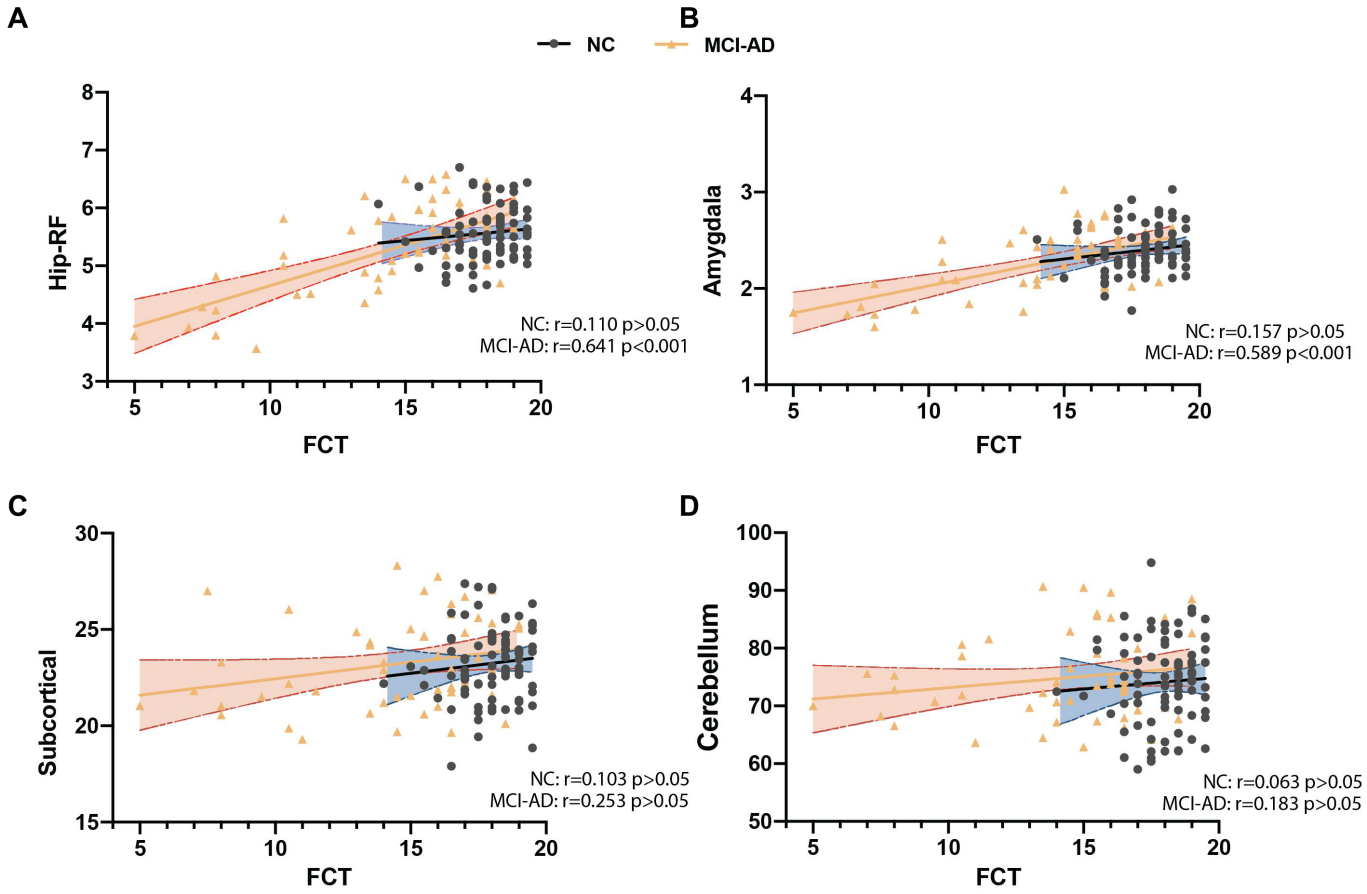
Correlation analysis between FCT scores and the volume of different brain regions in cognitive decline patient and subjects with normal cognition (NC). **(A)** FCT scores was significantly associated with the volume of hippocampus related fields (Hip-RF) in patient with cognitive decline but not in NC. **(B)** FCT scores was significantly associated with the volume of amygdala in patient with cognitive decline but not in NC. **(C,D)** Both in patient with cognitive decline and NC, FCT scores have no significant correlation with the volume of subcortical atlases of the striatum, globus pallidus and thalamus or the volume of cerebellum. Hip-RF: hippocampus related fields. Subcortical: subcortical atlases of the striatum, globus pallidus and thalamus (volume unit: cm^3^). NC: subjects with normal cognition (black and blue); MCI-AD: MCI and mild AD group (yellow and orange).

There were 135 participants (including 80 control subjects, 46 MCI and 9 mild AD patients) underwent brain MRI scanning, and we analyzed the correlation of total FCT score with the volume of four brain subregions. The results showed that FCT scores were positively correlated with the volume of hippocampal related fields (Hip-RF: *r* = 0.523, *p* < 0.001) as well as the volume of amygdala (*r* = 0.479, *p* < 0.001), but not with the volume of subcortical structures (*r* = 0.158, *p* > 0.05), or cerebellum (*r* = 0.070, *p* > 0.05; Figure 2). Since the hippocampus related regions and amygdala are believed to be closely related to learning and memory, we further conducted subgroup analysis to determine the correlation between FCT scores and the volume of cognitive-related regions both in NC subgroup and cognitive impaired populations, respectively. We found there was a more significant correlation between FCT scores and the volume of hippocampus-related regions as well as amygdala in the cognitive decline population (Hip-RF: *r* = 0.641, *p* < 0.001, amygdala: *r* = 0.589, *p* < 0.001). No correlation was found between the two indicators in the NC subgroup. In cognitive impaired subgroup, the volume of amygdala and hippocampus-related regions gradually decreases with the progress of the disease (Figure 3).

## Discussion

MMSE is a widely used screening test to quantitatively assess the severity of cognitive impairments in clinics (Folstein et al., 1975). It is usually be given in about 10 to 15 min and evaluates the global cognition with multiple domains (Tombaugh and McIntyre, 1992). It has a reliable sensitivity and specificity in differentiating patients with dementia from subjects with normal cognition (Folstein et al., 1975). However, an obvious shortcoming of MMSE is the ceiling effect or its poor sensitivity for distinguishing MCI which is attributed to a lack of complexity as well as the absence of executive function items (Trzepacz et al., 2015). Moreover, delayed recall scores make up 10% (3/30) of total MMSE scores which makes it insensitive in discovering subjects with episodic memory disorder in early stage.

FCT was designed as a quick, easily-used and reliable scale for testing cognitive impairment in multiple domains including episodic memory, language fluency, time orientation, visuospatial function, attention and executive function. The diagnostic power of FCT in discriminating patients with cognitive impairment from the subjects with cognitive function in normal range was reported to be effective with that of MMSE in previous cohort study (Zhang et al., 2019). By contrast with MMSE, the sub-score of episodic memory occupied 40% (8/20) in FCT total scores. The emphasis of memory makes FCT a prominent advantage in detecting episodic memory impairment over MMSE. In addition, the material of testing episodic memory in FCT was selected with eight culturally neutral pictures. This design facilitates the adoption of the FCT in countries with different cultural and educational background. Since the mean completing time for FCT was 5–6 min, it is especially suitable for apply in cognition screening in a large-scaled epidemiological studies (Zhang et al., 2019). Based on the above advantages highlighted by FCT, we conducted this study with an enlarged sample sized in the hope of promoting the clinical application of FCT.

Firstly, we analyzed the effects of sociodemographic and health-related factors on FCT and MMSE scores. The performance in FCT and MMSE decreased as the increase of age. Subjects with a higher education attainment performed better in FCT total scores, the sub-scores of delayed recall, language fluency, visuospatial ability as well as attention and executive function. The same results were observed in the association of education attainment with MMSE. These data added new evidence that high education has a protective effect for cognitive function (Rodriguez et al., 2019; Zamarian et al., 2020; Chen et al., 2021). The subjects who lived with family members performed better in FCT and MMSE than those lived alone, which indicating the family support acts a positive effect on cognition retain in old adults. Cheng GH found that moderate family support related to lower levels of depressive symptomatology and loneliness, which were associated with better cognitive function (Cheng et al., 2021). Several studies have also reported the association of older spouses’ lower cognitive function with the health of their partners (Gerstorf et al., 2009; Strawbridge et al., 2009) which suggested that individual cognitive ability and other health condition in older adults was deeply influenced by family members. The high BMI was positively associated with total FCT scores, sub-scores of delayed recall, language fluency, orientation of FCT as well as the sub-score of orientation of MMSE. Ntlholang et al. (2018) reported that BMI was positively related with immediate and delay memory, visuospatial/constructional ability, language and MMSE. Several cohort studies also demonstrated that high BMI had a decreased risk of cognitive impairment and underweight may be a significant risk factor for the cognitive impairment (Kim et al., 2020; Ren et al., 2021; Wu et al., 2021; Liang et al., 2022).

Secondly, we compared the correlation between the individual sub-scores of FCT or MMSE and a battery of neuropsychological tests in detecting specific domain of cognitive function. The AVLT including trials 1–5 total recall (AVLT total) and 30-min delayed recall (AVLT-delayed recall, N5), which is consistently considered as a sensitive and efficient method to assess the ability of word memory in elderly Chinese patients (Guo and Hong, 2001). Here we used the score of N5 (AVLT-delayed recall) to analysis the correlation with delayed recall sub-scores in FCT and MMSE. The AFT is a test for assessing language fluency which requires patients to name as many animals as possible within 60 s, with one point given for each unique name (Zhao et al., 2007). This is similar to the language fluency of FCT which used naming as many vegetables as possible. There was no significant correlation between AFT and the language sub-score of MMSE may partially attributing to the latter one containing naming, repeating, reading comprehension and writing abilities which covers a wider domain of assessing language. The BNT-30 (total score: 30 points) is one of the most widely used test to assess visual confrontation naming in clinical settings (Rabin et al., 2005) and the correlation of BNT-30 with MMSE seems much closer than that of FCT (Figure 1). Both the TMT-A/B (Lu et al., 2006) and SDMT (Smith, 1982) were widely used to assay the attention and executive function in old adults. The Rey–Osterrieth Complex Figure Test (CFT), a commonly used to assess the visuo-constructional ability and visual memory of neuropsychiatric disorders, including copying and recall tests (Guo and Hong, 2000). Here, we used the copy part of CFT to measure visuospatial perception ability. Our data showed that a higher correlation between FCT subitem scores with most of the specific neuropsychological tests that focus on specific domains of cognition than those of MMSE assessment. Together with the advantage of time-saving, it seems that FCT has excellent and convenient merits using as a broad-spectrum screening scale (Figure 1).

Thirdly, in order to explore whether the FCT scores is related to the brain structure, we analyzed the correlation between FCT scores and the volumes of different brain regions in populations with different cognition states. Interestingly, our research showed that in the total population, FCT scores positively correlated with the volume of the hippocampus-related regions and amygdala. Both the hippocampus related regions (Eldridge et al., 2000; Ystad et al., 2009; Kantarci et al., 2013) and amygdala (Cuenod et al., 1993; Cavedo et al., 2011; Poulin et al., 2011) are believed to be closely related to learning and memory. The subcortical structures and the cerebellum are generally considered to have little relationship with cognitive function, and we did not observe any correlation between FCT scores and the volume of these regions in the total population. These results aroused our interest, which suggesting that FCT scores might be a predict indictor of the volumes of cognitive-related brain regions. In further stratified analysis, there was a more significant correlation between FCT scores and hippocampus-related regions as well as amygdala volume in the cognitive decline population. However, no correlation was found between the two indicators in the cognitive unimpaired population. In cognitive decline population, the volume of amygdala and hippocampus-related regions gradually decreases with the progress of the disease (Jack et al., 2000; McDonald et al., 2012; Pini et al., 2016; Contador et al., 2021). Our results suggested that FCT might sensitively capture the volume changes of relevant brain regions during the course of memory decline. Therefore, as a primary screening scale, FCT might partially detect the neuropathological changes of AD. In future studies, we will explore the capacity of FCT scores to predict the longitudinal cognitive decline and the occurrence of AD.

Several limitations should be noted in the current study. First, the potential for selection bias exists since the participants included in this cohort have 6 years or more of education. Subjects with low education attainment should be included and the diagnostic power should be tested among those population in the future. Second, since pictured-based recall memory included in FCT, subjects with problems with visual disorders was restricted. The braille version of FCT should be developed in the future. Third, distinguishing MCI or mild AD with higher cognitive performance effectively from normal subjects is a leading and promising direction in the field of clinical research of AD. A larger sample size study containing participants with different education attainment need to be carried out in distinguishing early patients in the future.

## Conclusion

In the current study, FCT is further proved to be a quick, easily-used and reliable scale for detecting cognitive impairment in a community setting. Our data showed a significant correlation between FCT subitem scores and a comprehensive battery of neuropsychological tests that focus on specific domains of cognition. Moreover, FCT scores were significantly correlated with brain subregions related to memory but not subregions responsible for motor function. These findings lead to greater potential for adopting FCT in screening cognitive impairment in clinical research and practice.

## Data availability statement

The raw data supporting the conclusions of this article will be made available by the authors, without undue reservation.

## Ethics statement

The studies involving human participants were reviewed and approved by the Committee on Medical Ethics of Zhongshan Hospital, Fudan University. The patients/participants provided their written informed consent to participate in this study.

## Author contributions

XP, JZ and XC were responsible for subject enrollment. XP, XC, and YX performed the data analysis and wrote the paper. CZ and GF designed the whole study. XP, XC, JZ, YX, CZ, and GF agree to be accountable for all aspects of the work in ensuring that questions related to the accuracy or integrity of any part of the work are appropriately investigated and resolved. All authors have carefully reviewed this manuscript and involved in the drafting, critical revision and final approval of the manuscript for publication.

## Funding

This study was supported by grants from the National Natural Science Foundation of China (82171408 and 82171411), Medical Innovation Program of Fujian Health Committee (2020CXB049), and Medical and Health Technology Program of Science and Technology Bureau in Xiamen (3502Z20194028).

## Conflict of interest

The authors declare that the research was conducted in the absence of any commercial or financial relationships that could be construed as a potential conflict of interest.

## Publisher’s note

All claims expressed in this article are solely those of the authors and do not necessarily represent those of their affiliated organizations, or those of the publisher, the editors and the reviewers. Any product that may be evaluated in this article, or claim that may be made by its manufacturer, is not guaranteed or endorsed by the publisher.

## References

[ref1] 2022 Alzheimer's Disease Facts and Figures (2022). 2022 Alzheimer's disease facts and figures. Alzheimers Dement. 18, 700–789. doi: 10.1002/alz.12638 35289055

[ref3] Cavedo E. Boccardi M. Ganzola R. Canu E. Beltramello A. Caltagirone C.. (2011). Local amygdala structural differences with 3T MRI in patients with Alzheimer disease. Neurology 76, 727–733. doi: 10.1212/WNL.0b013e31820d62d9, PMID: 21339500 PMC3053328

[ref4] Chen G. Zhao M. Yang K. Lin H. Han C. Wang X.. (2021). Education exerts different effects on cognition in individuals with subjective cognitive decline and cognitive impairment: a population-based study. J. Alzheimers Dis. 79, 653–661. doi: 10.3233/JAD-201170, PMID: 33337379

[ref5] Cheng G. H. Chan A. Ostbye T. Malhotra R. (2021). Productive engagement patterns and their association with depressive symptomatology, loneliness, and cognitive function among older adults. Aging Ment. Health 25, 332–340. doi: 10.1080/13607863.2019.1686458, PMID: 31718250

[ref6] Contador J. Perez-Millan A. Tort-Merino A. Balasa M. Falgas N. Olives J.. (2021). Longitudinal brain atrophy and CSF biomarkers in early-onset Alzheimer's disease. Neuroimage Clin. 32:102804. doi: 10.1016/j.nicl.2021.102804 PMC8405839 34474317

[ref7] Cuenod C. A. Denys A. Michot J. L. Jehenson P. Forette F. Kaplan D.. (1993). Amygdala atrophy in Alzheimer's disease. An in vivo magnetic resonance imaging study. Arch. Neurol. 50, 941–945. doi: 10.1001/archneur.1993.00540090046009 8363448

[ref8] Eckerstrom C. Olsson E. Bjerke M. Malmgren H. Edman A. Wallin A.. (2013). A combination of neuropsychological, neuroimaging, and cerebrospinal fluid markers predicts conversion from mild cognitive impairment to dementia. J. Alzheimers Dis. 36, 421–431. doi: 10.3233/JAD-122440, PMID: 23635408

[ref9] Eldridge L. L. Knowlton B. J. Furmanski C. S. Bookheimer S. Y. Engel S. A. (2000). Remembering episodes: a selective role for the hippocampus during retrieval. Nat. Neurosci. 3, 1149–1152. doi: 10.1038/80671, PMID: 11036273

[ref10] Entis J. J. Doerga P. Barrett L. F. Dickerson B. C. (2012). A reliable protocol for the manual segmentation of the human amygdala and its subregions using ultra-high resolution MRI. NeuroImage 60, 1226–1235. doi: 10.1016/j.neuroimage.2011.12.073, PMID: PMC3665767 22245260

[ref11] Folstein M. F. Folstein S. E. McHugh P. R. (1975). Mini-mental state. A practical method for grading the cognitive state of patients for the clinician. J. Psychiatr. Res. 12, 189–198. doi: 10.1016/0022-3956(75)90026-6 1202204

[ref12] Gerstorf D. Hoppmann C. A. Kadlec K. M. McArdle J. J. (2009). Memory and depressive symptoms are dynamically linked among married couples: longitudinal evidence from the AHEAD study. Dev. Psychol. 45, 1595–1610. doi: 10.1037/a0016346, PMID: 19899917 PMC4203713

[ref13] Guo Q. C. L. Hong Z. (2000). Application of Rey-Osterrieth complex figure test in Chinese normal old people. Chin. J. Clin. Psych. 8, 205–207.

[ref14] Guo Q. L. C. Hong Z. (2001). Reliability and validity of auditory verbal learning test on Chinese elderly patients. J. Chin. Ment. Health. 15, 13–15.

[ref15] Jack C. R. Jr. Petersen R. C. Xu Y. O'Brien P. C. Smith G. E. Ivnik R. J.. (2000). Rates of hippocampal atrophy correlate with change in clinical status in aging and AD. Neurology 55, 484–490. doi: 10.1212/WNL.55.4.484, PMID: 10953178 PMC2724764

[ref16] Kantarci K. Weigand S. D. Przybelski S. A. Preboske G. M. Pankratz V. S. Vemuri P.. (2013). MRI and MRS predictors of mild cognitive impairment in a population-based sample. Neurology 81, 126–133. doi: 10.1212/WNL.0b013e31829a3329, PMID: 23761624 PMC3770173

[ref17] Kim G. Choi S. Lyu J. (2020). Body mass index and trajectories of cognitive decline among older Korean adults. Aging Ment. Health 24, 758–764. doi: 10.1080/13607863.2018.1550628, PMID: 30618275

[ref18] Liang F. Fu J. Turner-McGrievy G. Wang Y. Qiu N. Ding K.. (2022). Association of Body Mass Index and Plant-Based Diet with cognitive impairment among older Chinese adults: a prospective, Nationwide cohort study. Nutrients 14:3132. doi: 10.3390/nu14153132 35956314 PMC9370436

[ref19] Livingston G. Sommerlad A. Orgeta V. Costafreda S. G. Huntley J. Ames D.. (2017). Dementia prevention, intervention, and care. Lancet 390, 2673–2734. doi: 10.1016/S0140-6736(17)31363-6 28735855

[ref20] Lonie J. A. Tierney K. M. Ebmeier K. P. (2009). Screening for mild cognitive impairment: a systematic review. Int. J. Geriatr. Psychiatry 24, 902–915. doi: 10.1002/gps.2208 19226524

[ref21] Lu J. G. Q. Hong Z. Shi W. Lv C. (2006). Trail making test used by Chinese elderly patients with mild cognitive impairment and mild Alzheimer' dementia. Chin. J. Clin. Psych. 14:118.

[ref22] McDonald C. R. Gharapetian L. McEvoy L. K. Fennema-Notestine C. Hagler D. J. Holland D.. (2012). Relationship between regional atrophy rates and cognitive decline in mild cognitive impairment. Neurobiol. Aging 33, 242–253. doi: 10.1016/j.neurobiolaging.2010.03.015, PMID: 20471718 PMC2923665

[ref23] McKhann G. Drachman D. Folstein M. Katzman R. Price D. Stadlan E. M. (1984). Clinical diagnosis of Alzheimer's disease: report of the NINCDS-ADRDA work group under the auspices of Department of Health and Human Services Task Force on Alzheimer's disease. Neurology 34, 939–944. doi: 10.1212/WNL.34.7.939, PMID: 6610841

[ref24] Morris J. C. (1993). The clinical dementia rating (CDR): current version and scoring rules. Neurology 43, 2412–2414. doi: 10.1212/WNL.43.11.2412-a, PMID: 8232972

[ref25] Ntlholang O. McCarroll K. Laird E. Molloy A. M. Ward M. McNulty H.. (2018). The relationship between adiposity and cognitive function in a large community-dwelling population: data from the trinity Ulster Department of Agriculture (TUDA) ageing cohort study. Br. J. Nutr. 120, 517–527. doi: 10.1017/S0007114518001848, PMID: 30058503

[ref26] Petersen R. C. (2004). Mild cognitive impairment as a diagnostic entity. J. Intern. Med. 256, 183–194. doi: 10.1111/j.1365-2796.2004.01388.x 15324362

[ref27] Petersen R. C. Smith G. E. Waring S. C. Ivnik R. J. Tangalos E. G. Kokmen E. (1999). Mild cognitive impairment: clinical characterization and outcome. Arch. Neurol. 56, 303–308. doi: 10.1001/archneur.56.3.303 10190820

[ref28] Pini L. Pievani M. Bocchetta M. Altomare D. Bosco P. Cavedo E.. (2016). Brain atrophy in Alzheimer's disease and aging. Ageing Res. Rev. 30, 25–48. doi: 10.1016/j.arr.2016.01.002 26827786

[ref29] Poulin S. P. Dautoff R. Morris J. C. Barrett L. F. Dickerson B. C. (2011). Alzheimer's disease neuroimaging I. Amygdala atrophy is prominent in early Alzheimer's disease and relates to symptom severity. Psychiatry Res. 194, 7–13. doi: 10.1016/j.pscychresns.2011.06.014, PMID: 21920712 PMC3185127

[ref2] Rabin L. A. Barr W. B. Burton L. A. (2005). Assessment practices of clinical neuropsychologists in the United States and Canada: A survey of INS, NAN, and APA Division 40 members. Arch. Clin. Neuropsychol. 20, 33–65. doi: 10.1016/j.acn.2004.02.005 15620813

[ref30] Ren Z. Li Y. Li X. Shi H. Zhao H. He M.. (2021). Associations of body mass index, waist circumference and waist-to-height ratio with cognitive impairment among Chinese older adults: based on the CLHLS. J. Affect. Disord. 295, 463–470. doi: 10.1016/j.jad.2021.08.093, PMID: 34507227

[ref31] Rodriguez F. S. Zheng L. Chui H. C. (2019). Aging brain: vasculature I, behavior S. psychometric characteristics of cognitive reserve: how high education might improve certain cognitive abilities in aging. Dement. Geriatr. Cogn. Disord. 47, 335–344. doi: 10.1159/000501150, PMID: 31466060

[ref32] Sala I. Illan-Gala I. Alcolea D. Sanchez-Saudinos M. B. Salgado S. A. Morenas-Rodriguez E.. (2017). Diagnostic and prognostic value of the combination of two measures of verbal memory in mild cognitive impairment due to Alzheimer's disease. J. Alzheimers Dis. 58, 909–918. doi: 10.3233/JAD-170073 28527215

[ref33] Smith A. Symbol Digit Modalities Test. Western Psychological Services: Los Angeles, CA. (1982).

[ref34] Strawbridge W. J. Wallhagen M. I. Thai J. N. Shema S. (2009). The influence of spouse lower cognitive function on partner health and well-being among community-dwelling older couples: moderating roles of gender and marital problems. Aging Ment. Health 13, 530–536. doi: 10.1080/13607860802607330, PMID: 19629777

[ref35] Tombaugh T. N. McIntyre N. J. (1992). The mini-mental state examination: a comprehensive review. J. Am. Geriatr. Soc. 40, 922–935. doi: 10.1111/j.1532-5415.1992.tb01992.x 1512391

[ref36] Trzepacz P. T. Hochstetler H. Wang S. Walker B. Saykin A. J. (2015). Alzheimer's disease neuroimaging I. relationship between the Montreal cognitive assessment and Mini-mental state examination for assessment of mild cognitive impairment in older adults. BMC Geriatr. 15:107. doi: 10.1186/s12877-015-0103-3, PMID: 26346644 PMC4562190

[ref37] Tsoi K. K. Chan J. Y. Hirai H. W. Wong S. Y. Kwok T. C. (2015). Cognitive tests to detect dementia: a systematic review and meta-analysis. JAMA Intern. Med. 175, 1450–1458. doi: 10.1001/jamainternmed.2015.2152 26052687

[ref38] World Health Organization. Global Status Report on the Public Health Response to Dementia: Executive Summary. Geneva: World Health Organization. (2021).

[ref39] Wu S. Lv X. Shen J. Chen H. Ma Y. Jin X.. (2021). Association between body mass index, its change and cognitive impairment among Chinese older adults: a community-based, 9-year prospective cohort study. Eur. J. Epidemiol. 36, 1043–1054. doi: 10.1007/s10654-021-00792-y, PMID: 34370136

[ref40] Ystad M. A. Lundervold A. J. Wehling E. Espeseth T. Rootwelt H. Westlye L. T.. (2009). Hippocampal volumes are important predictors for memory function in elderly women. BMC Med. Imaging 9:17. doi: 10.1186/1471-2342-9-17 19698138 PMC2743662

[ref41] Zamarian L. Lenhart L. Nagele M. Steiger R. Gizewski E. R. Benke T.. (2020). Effects of cognitive functioning and education on later-life health numeracy. Gerontology 66, 582–592. doi: 10.1159/000510092, PMID: 32980844

[ref42] Zhang J. Wang L. Deng X. Fei G. Jin L. Pan X.. (2019). Five-minute cognitive test as a new quick screening of cognitive impairment in the elderly. Aging Dis. 10, 1258–1269. doi: 10.14336/AD.2019.0115, PMID: 31788337 PMC6844584

[ref43] Zhao Q. G. Q. Shi W. Zhou Y. Hong Z. (2007). Category verbal fluency test in identification and differential diagnosis of dementia. Chin. J. Clin. Psych. 3, 241–245.

