# Supplementary material for: A comparison of the five-minute cognitive test with the mini-mental state examination in the elderly for cognitive impairment screening
Source: Front Neurosci. 2023 Jun 9;17:1146552. doi: 10.3389/fnins.2023.1146552 (PMC10292014; doi:10.3389/fnins.2023.1146552)
Supplement: Supplementary file 1 [file Data_Sheet_1.docx]

**Supplemental Table 1. Correlation between the subitem scores of FCT or MMSE and specific neuropsychological assessments (n=333)**.

| **Variables** |  | ***r*** | **P** | **R^2^** |
| --- | --- | --- | --- | --- |
| **AVLT Delayed** | **FCT** | 0.441 | <0.0001 | 0.195 |
|  | **MMSE** | 0.361 | <0.0001 | 0.130 |
| **AFT** | **FCT** | 0.257 | <0.0001 | 0.066 |
|  | **MMSE** | 0.090 | ns | 0.008 |
| **BOSTON-30** | **FCT** | 0.195 | <0.001 | 0.038 |
|  | **MMSE** | 0.203 | <0.001 | 0.041 |
| **TMT-A** | **FCT** | -0.303 | <0.0001 | 0.092 |
|  | **MMSE** | -0.196 | <0.0001 | 0.039 |
| **TMT-B** | **FCT** | -0.360 | <0.0001 | 0.130 |
|  | **MMSE** | -0.227 | <0.0001 | 0.052 |
| **SDMT** | **FCT** | 0.311 | <0.0001 | 0.100 |
|  | **MMSE** | 0.192 | <0.0001 | 0.037 |
| **CFT** | **FCT** | 0.437 | <0.0001 | 0.191 |
|  | **MMSE** | 0.066 | ns | 0.004 |

Abbreviations: FCT: Five-minute cognitive test; MMSE: Mini-mental state examination; AVLT: Auditory Verbal Learning Test; AFT: Animal Fluent Test; BNT-30: Boston Naming Test-30; TMT-A: Trail Making Test A; TMT-B: Trail Making Test B; SDMT: Symbol Digit Modalities Test; CFT: Rey-Osterrieth Complex Figure Test.
